# Personal Characteristics for Successful Senior Cohousing: A Proposed Theoretical Model

**DOI:** 10.3390/ijerph19042241

**Published:** 2022-02-16

**Authors:** Pilar Monton, Luisa-Eugenia Reyes, Carlos-María Alcover

**Affiliations:** 1 International Doctoral School, Law and Social Sciences, Universidad Rey Juan Carlos, C/Quintana, 2-2^a^ Planta, 28008 Madrid, Spain; 2 Department of Business Management and Organization, Faculty of Business Economics, Universidad Rey Juan Carlos, P^o^ de los Artilleros 38, 28032 Madrid, Spain; luisa.reyes@urjc.es; 3 Department of Psychology, Faculty of Health Sciences, Universidad Rey Juan Carlos, Avda. Atenas s/n, Alcorcón, 28922 Madrid, Spain; carlosmaria.alcover@urjc.es

**Keywords:** senior cohousing, entrepreneurial intention, entrepreneurial competencies, psychological well-being

## Abstract

This paper aims to propose an integrated theoretical model with which to identify the personal characteristics, behaviors, and competencies of individuals who have successfully seen a senior cohousing project through to the residential stability phase. Numerous early-stage senior cohousing projects are registered each year. However, only a few of them are actually built, and when they are, the construction process takes an average of 10 years. Would-be cohousing residents have to put their tenacity and other competencies to the test to overcome the obstacles in their path before their residential complex is completed. The model proposed here analyzes senior cohousing initiatives as entrepreneurial undertakings. To this end, it draws upon a comprehensive review of the literature on entrepreneurship to identify the personal characteristics, behaviors, and competencies typical of entrepreneurs. In this model, participants in senior cohousing projects make use of these entrepreneurial competencies to help them overcome the obstacles to completing their housing development. However, for the would-be cohousing residents, the objective is not simply to build the residential complex but to enjoy the satisfaction and well-being this housing typology offers. Here, too, we find their entrepreneurial competencies can play a role.

## 1. Introduction

Retirement marks the end of a life stage, and more and more people consider it a good time to move home to improve their quality of life [1]. However, moving home may result in social relationships becoming more distant, and these ties may already have been weakened by retirement ending the daily social contact associated with the working environment [2]. Senior cohousing is an alternative housing arrangement that aims to meet the need for social contact and maintain a lifestyle in line with active-aging recommendations. In this model, people live in a collaborative group of neighbors who know one another and strive to maintain a sense of community [3] by building an environment more attuned to maintaining an active lifestyle than typical of the traditional idea of retirement [4]. However, although numerous early-stage projects are registered each year, the tasks involved in building and implementing a senior cohousing development often face numerous obstacles. These may lengthen construction times excessively, such that only 1 in 10 groups that start a project manages to realize their housing complex, and it is not uncommon for the process to take over 10 years [5].

This model aims to help identify the personal characteristics, behaviors, and competencies involved in successful senior cohousing initiatives to deepen our understanding of the personal factors underlying this successful outcome.

The success of entrepreneurial undertakings has been found to be related to personal characteristics [6,7,8,9], behavioral intentions [10,11,12], and entrepreneurial competencies [13]. Nevertheless, in the case of a senior cohousing project, completing the construction of the housing complex is not the final goal. The ultimate success of the initiative needs to be judged in terms of the cohousing residents’ reported well-being during the residential stability phase. Our literature review identified certain competencies that influence the perception of well-being from the collaborative lifestyle supported by this type of intentional community [14,15].

What is new in this study is that it analyzes senior cohousing initiatives as entrepreneurial undertakings with the ultimate goal of achieving well-being. While the personal characteristics, behavior, and competencies involved in entrepreneurship described by McClelland [13] are factors in the success of entrepreneurial initiatives, this theoretical model seeks to show that there is also a link between entrepreneurial competencies and the initial and final phases of the senior cohousing project. In the initial phase in which expectations are formulated, entrepreneurial competencies affect the formation of the intent described in Ajzen’s [10] theory of planned behavior. Later, in the final, residential stability phase, certain entrepreneurial competencies influence the perception of psychological well-being described by Ryff [16].

The main objective of this paper is to identify the personal characteristics, behavioral intentions, and competencies of people behind successful senior cohousing projects, i.e., those who manage to have their project built and then continue to enjoy the well-being this living arrangement can offer. It, therefore, starts with a review of the theoretical and empirical literature on the challenges faced by individuals who decide to embark on a senior cohousing project. This is followed by an analysis of the process of a senior cohousing venture, from the initial phase in which the intention to participate develops, through to the phase of residential stability. Thirdly, it proposes a theoretical model of analysis taking an integrated perspective to highlight the links between the personal characteristics, behavioral elements, entrepreneurial competencies, and the well-being outcomes achieved by people involved in a senior cohousing project who manage to see it through to the residential stability phase. Lastly, the paper ends with a discussion of the theoretical and applied implications and suggests some areas for future research.

## 2. Theoretical and Empirical Background

### 2.1. Senior Cohousing: Entrepreneurship Challenges and Barriers

Since its beginnings, the concept of cohousing has been shaped and reshaped by users of this housing typology and researchers into the phenomenon. Although each community is made to measure for its residents, there are certain features common to what is currently understood as a cohousing community. In general, the cohousing system is defined as a group of private dwellings located in a complex intentionally designed by its residents to meet their individual needs while facilitating social interaction [17]. Cohousing is a form of intentional community that emerged in the 1970s in Denmark and the Netherlands [18,19]. Senior cohousing communities are not substantially different from intergenerational complexes in terms of how they are created and structured. Both typologies aim for a balance between private and community life; however, in the case of senior cohousing, the emphasis is on a lifestyle attuned to its residents’ potential needs in view of their age range [20]. Although the senior segment initially only accounted for a small share of these projects, it subsequently expanded considerably [18]. Over the years, senior cohousing progressively spread to the United States, Australia, Canada, and other European countries in both northern (e.g., Germany, Sweden) and southern Europe (e.g., France, Spain) [21,22]. Since this lifestyle can significantly improve older people’s quality of life by enhancing personal autonomy, social support, and community solidarity [23], this type of community-living initiative has attracted interest and gradually expanded its presence across much of the world [21,22,23].

Since this housing typology emerged in northern Europe in the early 1970s, numerous researchers have studied it. In 2000, Vestbro [24] compiled a review of theoretical and empirical studies of collective housing and cohousing published so far. Studies and publications have since proliferated, with various approaches being taken to the subject, including guides for people interested in undertaking this kind of project, such as Durrett’s *The Senior Cohousing Handbook* [20], as well as research into the effect the sense of belonging to a community has on cohousing residents’ well-being [25]. In 2016, Tummers [26] published a comprehensive review of the literature on the cohousing movement and classified publications into the following five groups:

1. Empirical studies and publications by residents of cohousing developments themselves;
2. Research focusing on demographic change and the evolving social model, with the shift away from traditional family structures, in the quest for well-being based on independent and autonomous aging;
3. Studies looking at architectural design and how it contributes to social interaction;
4. Research focusing on neighborhoods and sustainable urban development;
5. Emerging topics concerning the legal and financial aspects of cohousing.

Despite the wealth of publications examined, Tummers noted that each discipline has a different conceptualization of the cohousing phenomenon, creating the need for a solid body of knowledge with which to explain why many initiatives founder and only a few reach fruition [26]. In the same vein, Boyer and Leland [27] analyzed the contradictions in a model that, on paper, looks attractive to many potential participants, given its collaborative nature and the way it is designed to enhance social contact, but whose slow growth suggests it is a housing option available only to a few [27].

The group involved in a senior cohousing initiative participates in all the phases of the design and construction of the housing complex and, subsequently, handles its management [5]. As in any other type of undertaking, from the outset, they will have to overcome the obstacles that they meet before the housing complex is successfully built. Obstacles identified in the literature that are of particular relevance to this analysis from an entrepreneurial perspective include those affecting group cohesion [28], understanding of legal requirements [28], the balance between the economic and functional viability of the project [3], and designing the housing complex and coordinating its construction [29]. 

Group cohesion is relevant because the land purchase and construction work are processes requiring collective agreements on the general geographical location and the choice of the specific plot [30]. Interactions and negotiations with the planning authorities and other stakeholders may often prove slower than desired and if the group of future residents is not sufficiently cohesive, some original members may drop out [28].

A degree of familiarity is needed with the legal and regulatory requirements at all the stages of the project, particularly an understanding of the legal framework applicable to the housing complex [31]. Understanding the national and local laws applicable to the intended location, regulations concerning utility connections, and other issues relating to the future development of the land, are sometimes insurmountable challenges for a population group that has never previously had to tackle issues of this kind. This situation has drawn criticism, with demands for new regulations to facilitate these processes and coordinate interactions with the authorities [28].

In terms of the balance between the economic and functional viability of the development, a cohousing complex needs to be large enough to ensure economic viability and a rich network of neighborly relations, but not so large as to slow down decision making [20]. Accordingly, the optimal number of housing units in the complex tends to lie in the range of 15 to 30 apartments [20]. Additionally, not all members of cooperatives have the same financial resources, making it necessary to obtain financing. Therefore, group members need to meet a series of solvency and liquidity requirements at a point in their lives when their income often derives from their retirement pension [32].

When it comes to designing the housing project, even after finding the land on which to build it, defining the legal structure, and obtaining finance, the spatial design of the housing complex can also be a challenge for the group members. To a large extent, the intended communal activities will determine the number and arrangement of both the individual units and shared spaces [33]. In planning the housing complex, the role of the architect is usually limited to the formal design, informed by the opinions of the community’s future residents. The group’s involvement in this task allows them to design the housing to meet their present or future needs. Workshops allow the group to exchange views on topics such as accessibility, the design of the homes and communal areas, or the design of spaces for leisure activities and community tasks. This aligns senior cohousing more closely with the concept of active aging than with the traditional idea of retirement [34]. There is a positive relationship between collective self-build and social cohesion [35] and the cohousing residents’ intention to live together as active, mutually supportive participants in their community of neighbors creates a shared environment that fosters communication, the exchange of skills, and continuous learning [36]. 

Although active participation in the design process is considered beneficial for group cohesion, it can also be a constraint when excessive iterations make it hard to meet construction deadlines [29].

Participation in the design of the cohousing project from the outset appears to have many motivating features, with its promise that residents will maintain a close community life while keeping their independence and privacy. However, its downside is that it can hinder or lengthen the time taken to complete the construction project, testing the competencies and determination of the would-be cohousing residents [26].

### 2.2. Characterization of the Senior Cohousing Entrepreneurs

The initiative’s entrepreneurs will have to put their tenacity and other competencies to the test to overcome the obstacles in their path before their residential complex is completed [26]. Typical competencies of entrepreneurs include a combination of personal characteristics, as well as skills and knowledge acquired from experience. These competencies include vision [37], ability to set goals [38], proactive personality [39], tenacity [40], and perceived self-efficacy [41]. However, the willingness to become involved in a venture will also be affected by socio-demographic and human capital factors, the most frequently cited of which are gender, age, marital status, level of educational attainment, employment situation, and income [42]. These factors take on special relevance in the case of senior cohousing entrepreneurs, as the stereotypes associated with the socio-cultural environment in which potential entrepreneurs find themselves can influence their perceptions of the likelihood of whether they are able to respond successfully to events [43]. 

Advances in health care, along with improved living conditions and healthy habits, have made it possible to overcome the outdated image of workers reaching retirement age in poor physical condition. Today, most people reach retirement in good health and are able to participate in activities benefiting society and themselves [44]. They are also able to draw upon their accumulated life experience, which provides them with valuable human capital built up from both formal and informal education, practical learning acquired in the workplace, and abilities deriving from their experience [45]. With this human capital, individuals who have confidence in their capabilities to carry out a project have a stronger predisposition to participate when decisions are made [41], such that their belief in control will shape the process from the outset [46].

One of the key aspects of control beliefs, in addition to individuals’ belief in their own abilities, is the locus of control. This is defined in terms of the belief individuals hold regarding the factors controlling their lives. Rotter [47] defined individuals to have an internal locus of control when they have a predominant tendency to feel responsible for their successes and failures. By contrast, individuals with an external locus of control will attribute outcomes to external forces—luck, life, fate, or any factor they believe they are unable to influence [47]. People with a strong internal locus of control will tend to take a more active role in relation to events and take responsibility for the results obtained [48]. This is the attributional style that has been found to predominate among entrepreneurs [9].

However, even potential entrepreneurs with a high internal locus of control and confidence in their own abilities will need to undergo a preparatory process in which they analyze whether they want to participate in the project [10]. The different theories and models for studying how entrepreneurial intention forms describe entrepreneurship as a process that needs time to evolve and develop, while the individual decides if they are interested in investing the energy and effort necessary to carry out the project [49]. Back in 1975, Fishbein and Ajzen [50] proposed the theory of reasoned action, in which two types of factors determine the intention that leads to a behavior. The first factor is the individual’s attitude to the project, their expectations, and how important the expected results are. The second set of variables concerns subjective norms, consisting of social norms, the perceived opinion of the people around them, those close to them, or role models. In a subsequent study to his development of the theory of reasoned action, Ajzen [10] put forward the theory of planned behavior (TPB), adding the dimension of the individual’s perception of control to the preceding model. This refers to the extent to which individuals feel they have the necessary resources to carry out the planned project. Individuals’ intention to undertake the project will be weakened when they believe they lack the resources to carry it through with a sufficient likelihood of obtaining the desired results. Conversely, their intention will be strengthened when they perceive the project to be viable because they consider themselves to have the competence and specific capabilities necessary to carry it out successfully [51].

The theories described do not refer to the point in time at which individuals turn their intention into the action of undertaking a project. However, in their entrepreneurial event model, Shapero and Sokol [11] in fact include this factor, situating the origin of the formation of the entrepreneurial intention at the moment at which there is a triggering event, which will be perceived by the potential entrepreneur as an opportunity to act proactively, precipitating the decision-making process that leads to the entrepreneurial action. Building on the Ajzen and Shapero–Sokol models, Krueger and Brazeal [12] developed their model of entrepreneurial potential, integrating the concepts of desirability and perception of feasibility, Ajzen’s TPB, together with Shapero and Sokol’s triggering event. Although there are differences between the three models, they share the relevance the authors attribute to the perception of feasibility. Potential entrepreneurs’ confidence in their own capabilities is not just a powerful predictor while making the decision to undertake the project, but it also has a positive influence on its subsequent success [52].

As regards the entrepreneurial competencies that have the strongest influence on the various phases of the project, there are two streams of research. One holds that certain competencies will only affect the entrepreneurship process during the initial phase in which the individual decides to participate in a project, and other competencies will prove to be of more importance in achieving a successful project outcome over the longer term [53]. Nevertheless, Rauch and Frese [52], while noting that the success of entrepreneurial projects will also depend on other external factors, demonstrate that there are personal characteristics and competencies such as motivation to achieve, a proactive personality, and self-confidence, that have an influence both on the decision to take part in the project and on the success obtained once it is underway [52].

For Veciana [54], the process of entrepreneurship does not end with setting up the project after the gestation of the idea and project creation. Rather, the launch phase entails a period of adaptation and readjustment, a change in the individual entrepreneur’s role that will prepare them for the achievement of the objectives that were the purpose of project gestation. In contrast to the case of a business venture, in which the objectives may be economic profit and growth, in the case of a senior cohousing project, after the initial launch, the objectives will be to achieve a perception of well-being and satisfaction with the lifestyle in the housing complex [19]. The majority of older people who chose to participate in the project and live in a senior cohousing complex report positive experiences from mutual assistance and solidarity [23]. Nevertheless, although more attention tends to be paid to the advantages, research also shows that for some residents it may be a challenge to strike a balance between private and communal life [19]. Consequently, not all participants report the same degree of well-being in the close communal living and lifestyle offered by the senior cohousing model [36].

## 3. Integrated Theoretical Model: Entrepreneurship and Well-Being in Senior Cohousing

**Figure 1:**
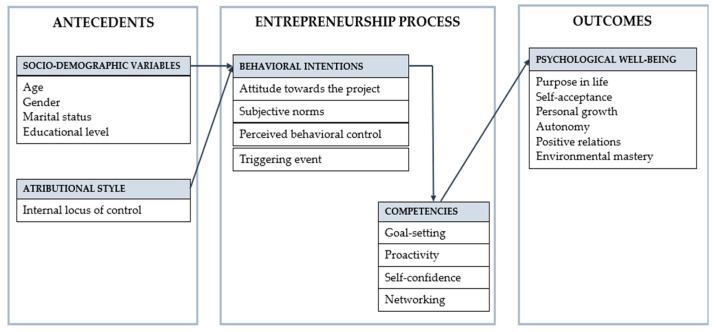
Theoretical model proposed on the senior cohousing entrepreneurship.

In what follows, a proposed analysis of entrepreneurship in senior cohousing is presented building on the theoretical and empirical foundations presented above. As shown in Figure 1, the proposed model has three component parts: (1) antecedents (socio-demographic variables and attributional style); (2) entrepreneurship process (behavioral intention and competencies); (3) outcome (psychological well-being). The model, therefore, integrates the main variables involved in the different phases of a senior cohousing project, from the individual’s forming the intention to participate, through to subsequent success with the materialization of the perception of well-being during the phase of residential stability. The proposed model does not claim to offer an exhaustive account of the variables that may influence the process as a whole. Following the principle of parsimony, the objective of this integrated model is to include those variables that empirical research has shown to be more specific and relevant to senior cohousing entrepreneurship.

### 3.1. Antecedents: Socio-Demographic Variables and Attributional Style

Researchers in the field are divided as to the influence of age on entrepreneurship. Some scholars argue that older entrepreneurs will have the benefit of experience on their side, a sounder financial position, and fewer family responsibilities [6]. Others, however, maintain that young people have the advantages of more energy, more up-to-date knowledge, and are more open minded [55].

As regards gender differences in entrepreneurship, recent research frequently mentions higher levels of self-confidence among men than women, while women perceive the environment to be more challenging and receive less approval from their close relations, which has a significant negative impact on women’s entrepreneurial orientation [7].

Researchers in various areas have analyzed the impact of marital status on entrepreneurship, whether starting and growing a business by taking on employees or for self-employment [56]; however, there are no reported findings on the influence of marital status on decision making on cohousing projects.

The model’s socio-demographic variables include the level of educational attainment. A higher level of education is generally associated with the development of skills such as a capacity for analysis. This can contribute to the development of opportunities and boost individuals’ self-confidence when they come to undertaking projects [8]. However, there is no consensus in the research community as to how decisive this factor is, given that successful entrepreneurship calls for numerous other characteristics, which may be more relevant [57].

In terms of attributional style, the impact of an internal locus of control is included, based on prior research showing its significant role on entrepreneurial behavior [9,47]. Individuals who feel they are unable to control outcomes have little motivation to actively change their environment and start projects [9]. Individuals with high values for their external locus of control, therefore, tend toward passivity. However, when individuals perceive a situation as being the outcome of their individual behavior, i.e., they have an internal locus of control, they take a more active role and feel responsible for the results obtained [48].

### 3.2. Entrepreneurship Process: Behavioral Intentions and Competencies

The application of Ajzen’s TPB [10] to the process of formation of entrepreneurial intention includes a phase in the process in which the individual evaluates the expected satisfaction that would be derived from the successful completion of the project. However, this will not be the only factor influencing an individual’s entrepreneurial intention. Individuals also assess whether they believe they possess the necessary capabilities and resources, and both aspects—their attitude toward the project and their perceived self-efficacy—may be influenced by the opinions of those around them, based on which they build their subjective norms [10]. The model of the entrepreneurial event proposed by Shapero and Sokol [11] introduces the idea of a triggering event, to which the model developed by Krueger and Brazeal adds entrepreneurial potential [12]. The combination of the three models outlined above, in the context of the analysis of the intention to participate in a senior cohousing project, will cover the following aspects: attitude toward the project, subjective norms, self-efficacy, and the triggering event for the intention.

In the case of a senior cohousing initiative, we assume that the more attractive the intended objectives of potential involvement are, and the higher importance subjects place on each of these goals for their future well-being, the more positive their attitude toward the project will be. Following the TPB [10], subjective norms refer to the opinions that subjects perceive from the people around them about their involvement in the project and reflect the degree of influence these opinions have on their intention to participate. Self-efficacy will be defined as the individuals’ perceptions of their own capabilities and resources to address the obstacles that may arise before the project is completed. As regards the triggering event behind the intention, reaching retirement could trigger the entrepreneurial intention when older adults perceive this situation as an opportunity to take charge and determine where and with whom they want to live in the future.

In terms of competencies, following the work of McClelland [13], researchers in various areas concur on the various entrepreneurial competencies concerned such as a vision of the future, initiative, a proactive attitude, the need for independence, moderate risk aversion, motivation to achieve, and self-efficacy [58]. Of these competencies, as will be argued below, those included in the proposed model are expected to be positively related to both the success of the initiative after the entrepreneur has taken the decision to participate in the project and the final success of the project in the form of participants’ perceived well-being and satisfaction.

For McClelland [59] vision and goal-setting ability mean the individual entrepreneur needs to know how to define their objectives clearly [13]. The goal-setting theory formulated by Locke in 1968 [60] argued that individuals who consciously set specific goals increase their motivation by identifying the goal as an action with a purpose, such that the performance they can achieve, as well as their level of commitment to the project, would be higher in the case of complex targets requiring a certain level of excellence [61]. 

A proactive attitude is characterized by a quest for new opportunities and answers [62]. Individuals who act proactively are in something of a state of alert, in terms of both responding to problems and staying abreast of events, and exploiting opportunities before others around them have identified potential new ways of achievement [63]. This attitude is a prerequisite for the effective formation of intention in several models of entrepreneurial intention. For instance, Shapero and Sokol’s entrepreneurial event model [11] includes the existence of a triggering event and a “propensity to act” as the starting points of the entrepreneurial process. Similarly, the entrepreneurial potential model developed by Krueger and Brazeal [12] involves the idea that entrepreneurial potential turns into effective intention when a specific event is identified by an entrepreneur as an opportunity to act proactively. In the case of cohousing, the date of retirement may act as the triggering event for senior cohousing residents’ intentions, alerting them and triggering their proactive behavior to take control over how they want to spend their retirement. 

McClelland’s studies [59] ascribe entrepreneurial individuals a high level of self-confidence, which he terms independence and self-confidence, and a perceived ability to respond effectively to unforeseen circumstances. They are also able to analyze other people’s opinions dispassionately and are not easily upset by opinions contrary to their own. These are circumstances in which individuals will strengthen their behavioral intention, perceiving it to be feasible as they consider themselves able to carry it out successfully [64].

For the entrepreneurs described by McClelland, the ability to maintain a rich network of contacts is a competency that translates into a good source of knowledge and facilitates the gathering of potentially relevant information for the achievement of their entrepreneurial goals [65]. The necessary decision making before starting an entrepreneurial project occurs in a context of uncertainty, and entrepreneurs will want to discuss their ideas with people whose opinions they value, hence the advantage of having contacts to draw upon for input [66].

### 3.3. Outcomes: Psychological Well-Being

One of the most widely used models for analyzing well-being is the construct defined by Ryff [67], which postulates the existence of six independent dimensions of psychological well-being: purpose in life, self-acceptance, personal growth, autonomy, positive relations with others, and environmental mastery. In relation to the psychological well-being of older adults, Ryff [16] reviews the approach taken by other researchers [68], who she considered having based their analysis on certain negative features of aging, without paying sufficient attention to the positive aspects successful aging can have for older people. 

The dimensions proposed by Ryff subsequently became the object of study by numerous members of the research community, who have deepened our understanding of them and the relations that link them to individual attitudes and competencies. For Seligman [14], maintaining a sense of purpose in life and being able to set goals consistent with it are the basic pillars of well-being. Researchers into psychological well-being have, therefore, focused their attention on the positive reinforcement individuals obtain when they invest effort in exploiting their own potential and developing their capabilities, finding that, in optimal circumstances, when the targets and purpose in life are supported by the individual’s strengths and virtues, they work together to promote personal growth [69].

A proactive personality is a key entrepreneurial competency. Entrepreneurs influence their environment by identifying the opportunities that arise and acting accordingly [9]; proactive individuals tend to be agents of change in their lives rather than let themselves be led by environmental forces. Their intentional activities will sustain or enhance their perception of well-being [15]. People who report a high degree of environmental mastery feel able to choose or influence their surroundings, which gives them a sense of control over their everyday activities [70]. Their interventions on their surroundings will enable them to influence their environment, and their achievements will produce a satisfying perception of a reciprocal interplay of intrapersonal, behavioral, and environmental determinants [71]. 

Regarding the competency of self-confidence, which McClelland [59] refers to as independence and self-confidence might suggest he describes an individualistic profile. However, researchers into the emotional intelligence construct have shown that entrepreneurs who have a high level of self-confidence also demonstrate high levels of competencies relating to teamwork and collaboration [72]. People showing self-confidence resist social pressures better by relying on their convictions while self-regulating their behavior better than individuals who feel their standing and personal authority is being threatened [73]. Self-confident individuals must have previously undergone a self-evaluation to perceive their strengths and weaknesses, maintaining a positive attitude toward both aspects, accepting them as an integral part of them [74], which also means an implicit acceptance of their life history [75].

## 4. Discussion

One common feature of senior cohousing arrangements mentioned in the specialist literature is the participatory process of building design [20]. However, there is a less detailed description of the preliminary and subsequent activities future cohousing residents need to undertake efficiently in order to complete their project. These activities involve everything from understanding local legislation on construction features, working in partnership with architects on the design of the building plans, arranging sources of finance, negotiating with local authorities over utility connections, and drawing up the rules for the internal functioning of the community [28]. 

Analyzing senior cohousing initiatives as entrepreneurial projects that entail an initial phase in which individuals decide to participate, along with the design and development phase to see the construction through to completion, will yield information about the competencies of participants who have managed to realize their housing development through to its operational phase. The main contribution of the proposed model is to fill the gap in senior cohousing research by providing an integrated framework that includes antecedent variables, process variables, and well-being outcomes. Additionally, empirical confirmation of the model will enable relationships to be established between the variables in the process and to make predictions as to their efficacy, as well as identify the possible influence of other mediating or moderating variables specific to this kind of entrepreneurial behavior and its outcomes.

However, the ultimate goal extends beyond completing the construction work and seeing the senior cohousing complex through to its operation. The project will only be a success if it brings well-being for the group’s participants during the residential stability phase. For many years, gerontology has sought to nuance the stereotype of old age as a time of solitude [76], although individuals who are unable to maintain their social network might lose contact with people they have established ties with through work. Even so, although social isolation does not necessarily result in feelings of loneliness, the two concepts are interrelated. Social isolation refers to an objective situation, voluntary or otherwise, characterized by a scarcity of social relations, whereas feelings of loneliness concern the sense of dissatisfaction with which individuals perceive a lack of social contact or the poor quality of social interactions [77]. Faced with a situation that may objectively be classed as social isolation, some people experience profound feelings of loneliness, whereas others perceive none at all [78]. In the case of individuals with a moderate ability to maintain a social network, once they have adapted to retirement, they would seek to establish new relationships as a matter of course through activities of interest to them. Thus, after a certain period, they can be expected to have re-established several social contacts they may consider sufficient and attuned to the lifestyle they have chosen to adopt [76]. 

The debate over the complex balance between interdependence and individualism remains ongoing [79]. Cohousing may offer a form of residential environment that is attractive to individuals looking for a sense of belonging to a community and who feel that today’s urban lifestyles do not foster mutual support [80]. However, some people also argue that the type of relationship created in a cohousing community is closer to that of a traditional community and is not always desirable for those who may sometimes choose not to interact with fellow residents because they often prefer privacy [81]. Consequently, not everyone can be expected to increase their perception of well-being by living in a senior cohousing complex rather than in an ordinary neighborhood. The shared common areas and active social and community life typical of this model of living arrangement may represent a more satisfactory lifestyle for individuals with the skills to establish positive relations with others and maintain their social network, and for whom the tasks needed in the complex are a source of well-being, as they provide meaning through work caring for neighbors and benefiting the community [36].

### 4.1. Future Directions

More empirical research is, therefore, needed to analyze this area of study in more detail to enrich and broaden the model and thereby help define more precisely the competencies related to the feasibility of cohousing projects, such that the analysis and dissemination of success factors from previous experiences can serve as a form of knowledge transfer to future entrepreneurs in senior cohousing projects.

### 4.2. Limitations

Despite its contributions, the proposed model has some limitations that require further development. The main limitation refers to the number of variables included since our proposal only considers individual variables. Other factors can probably also exert some influence, especially in the case of antecedents and entrepreneurial process variables. For example, an expanded model could include contextual variables, such as levels of environmental uncertainty, economic crises or recessions, or unpredictable local or global disruptions, as has been the case in the last two years with the COVID-19 pandemic. In particular, how these contextual variables interact with individual variables should be analyzed, both in the antecedents and during the entrepreneurship process. In addition, a broader model should also include other possible outcomes of participating in senior cohousing, such as physical and mental health status, or indicators of satisfaction and quality of life. Despite these limitations, the proposed model represents a significant step forward in understanding cohousing initiatives for mature and older people, a booming social, economic, and life activity still under-researched. Our model aims to achieve a heuristic value to guide future research in this field.

## 5. Conclusions

To summarize, the main contribution of this study is to offer an integrated model with which to analyze the personal characteristics, behavioral intentions, and competencies of the individuals who manage to put in practice a senior cohousing project and subsequently live the lifestyle this housing typology offers with satisfaction. This theoretical model is based on variables considered fundamental to the solidity of the proposal, given that they have been analyzed in previous studies not focusing specifically on senior cohousing. However, it may subsequently be worth including other competencies or aspects that enhance the analysis of the profile of individuals who manage to complete the construction of a senior cohousing complex and live out their retirement satisfied with this form of collaborative living.

## Data Availability

Not applicable.
